# Gastrointestinal stromal tumor with intracranial metastasis: case presentation and systematic review of literature

**DOI:** 10.1186/s12885-019-6316-7

**Published:** 2019-11-15

**Authors:** Marc Prablek, Visish M. Srinivasan, Aditya Srivatsan, Stephanie Holdener, Mazen Oneissi, Kent A. Heck, Ali Jalali, Jacob Mandel, Ashwin Viswanathan, Akash J. Patel

**Affiliations:** 1 Departments of Neurosurgery, 7200 Cambridge, Suite 9A, Houston, TX 77030 USA; 2 Pathology & Immunology, One Baylor Plaza, BCM 315, Houston, TX 77030 USA; 3 Neurology, Baylor College of Medicine, 7200 Cambridge, 9th Floor, Houston, TX 77030 USA; 4 University of Texas MD Anderson Cancer Center, Houston, TX USA; 5 Jan and Dan Duncan Neurological Research Institute, Texas Children’s Hospital, Houston, TX USA

**Keywords:** Gastrointestinal stromal tumor, GIST, GIST intracranial metastasis, GIST brain metastasis

## Abstract

**Background:**

Intracranial metastasis of Gastrointestinal Stromal Tumors (GISTs) is rare but presents unique treatment challenges. We present a case of intracranial metastasis of GIST with a systematic review of the literature. A literature search using key terms “‘gastrointestinal stromal tumor’ AND brain AND metastasis”” was conducted through May 2019 via Embase and Pubmed according to PRISMA guidelines. Only cases describing intradural metastases rather than calvarial or intraorbital metastases were included.

**Case presentation:**

A 57-year-old woman with history of GIST metastatic to the liver presented with a six-week history of left facial weakness, left hearing loss, and left facial numbness, and a one-week history of headaches, gait disturbance, and dizziness. MRI revealed a contrast-enhancing dural-based left middle cranial fossa mass measuring 2.9 cm × 3.1 cm × 3.4 cm with extension into the internal auditory canal and cerebral edema. A left temporal craniotomy was performed to excise the lesion, and the patient was discharged to a rehabilitation facility at her preoperative baseline. Intraoperative pathology revealed a spindle cell neoplasm, postoperative MRI demonstrated gross total resection of the lesion, and microscopic analysis demonstrated sheets of spindled tumor cells with short ovoid, irregular, hyperchromatic nuclei and scattered large atypical nuclei without extensive necrosis. Immunohistochemical staining was positive for KIT proto-oncogene (CD117, c-KIT), and the patient was put on imatinib (400 mg/day).

**Conclusions:**

Of the 18 cases analyzed and our present case, metastasis typically involved the cerebrum with only one in infratentorial elements. The tumors in seven of the cases involved the dura, and one case metastasized to the pituitary. Eight patients died following treatment. Surgery remains the mainstay of intracranial metastatic GIST, however there are many reports of good responses to radiation or chemotherapy alone. More investigation is required to determine the best treatment course for these patients.

## Background

Gastrointestinal Stromal Tumors (GISTs) are the most common mesenchymal neoplasms of the gastrointestinal tract, arising from the Interstitial Cells of Cajal. The most common location is the stomach (60%), followed by the jejunum and ileum (30%), duodenum (5%), and colorectum (< 5%), with rare reported cases arising in the esophagus, appendix, and gallbladder. Initially thought to be tumors of smooth muscle, advances in immunohistochemistry and molecular genetics have found that this is not the case. Specifically, most GISTs express KIT (CD 117, c-KIT), a highly sensitive and specific marker for GIST, while leiomyomas and leiomyosarcomas do not [1]. Anoctamin 1 (ANO1, DOG1), is another tissue marker that can be useful in the diagnosis of GIST [2]. GISTs are largely benign, but up to 30% may have malignant behaviors determined by the mitotic rate, primary site, size, and metastasis, most commonly to the liver, lung, and bone [1, 2]. Intracranial metastasis is rare and presents unique treatment challenges.

Imatinib, the mainstay medical treatment for GIST, does not cross the blood-brain barrier, and as such may offer limited clinical benefit to patients with intracranial metastasis [3]. Nevertheless, some authors have reported success in the treatment of intracranial GIST metastasis with imatinib [4]. Sunitinib, another tyrosine kinase receptor antagonist, is sometimes used as a second-line therapy in patients with metastatic GIST [5]; however, its role in the management of intracranial metastasis remains undetermined. Radiotherapy for metastatic GIST has not been definitively shown to be effective [1], as they are generally considered radioresistant, but little data exists regarding the treatment of intracranial GIST metastases with radiation. Despite this, some series have proposed a role for radiation therapy, especially for tumors that are surgically unfavorable [3].

Here, we present a case of intracranial metastasis of GIST with a discussion of relevant literature regarding this rare clinical scenario. We aim to study our current case with respect to historical studies to better investigate the best course of treatment for patients with this rare manifestation of GIST.

## Methods

A thorough systematic review of the literature was performed through the databases Embase and Pubmed from dates of inception to May 2019 to identify cases of GIST metastatic to the brain. The string “‘gastrointestinal stromal tumor’ AND brain AND metastasis”” was used as either keywords or Medical Subject Headings terms to identify all eligible studies. Inclusion and exclusion criteria from the Preferred Reporting Items for Systematic Reviews and Meta-Analyses (PRISMA) guidelines were used to vet the retrieved articles [6]. For the purposes of this review, cases were included only if they described intradural metastases rather than calvarial or intraorbital metastases. Abstracts, conference presentations, and editorials were excluded. As there is a paucity of cases in the literature, cases were included regardless of date of publication, source of publication, or inclusion in previous literature reviews. Studies were reviewed by a single reviewer (M.P.), and the senior investigator (A.P.) reviewed the final results. Data regarding demographics, modality of treatment, and treatment response were recorded for each case where available.

## Case presentation

**Fig. 1:**
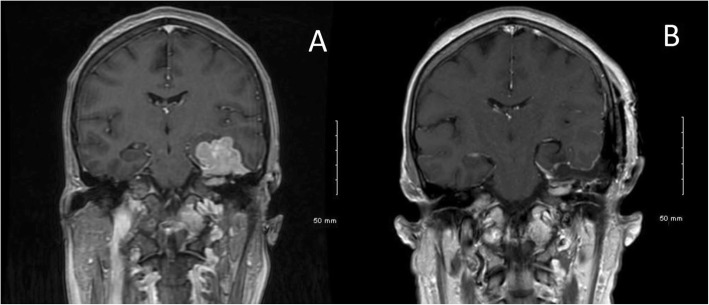
Representative T1-weighted post-contrast images preoperatively (**a**) and postoperatively (**b**). Pre-operative (**a**) and post-operative (**b**) coronal MRI, T1-weighted-post contrast. A lobulated, homogenously enhancing lesion is seen to be arising from the dura of the middle fossa and tentorium and compressing the temporal lobe. Gross total resection is noted in the post-operative image

A 57-year-old woman with known history of biopsy-proven GIST metastatic to the liver presented with a six-week history of left facial weakness, difficulty closing her left eye (House-Brackmann 4), complete left hearing loss, and left facial numbness. She had been previously diagnosed at an outside institution and initiated on imatinib (400 mg daily) 3 months prior to presentation, In the week prior to presentation, she had developed headaches, gait disturbance, and dizziness. MRI revealed a contrast-enhancing dural-based left middle cranial fossa mass measuring 2.9 cm × 3.1 cm × 3.4 cm with extension into the internal auditory canal and associated cerebral edema (Fig. 1).

**Fig. 2:**
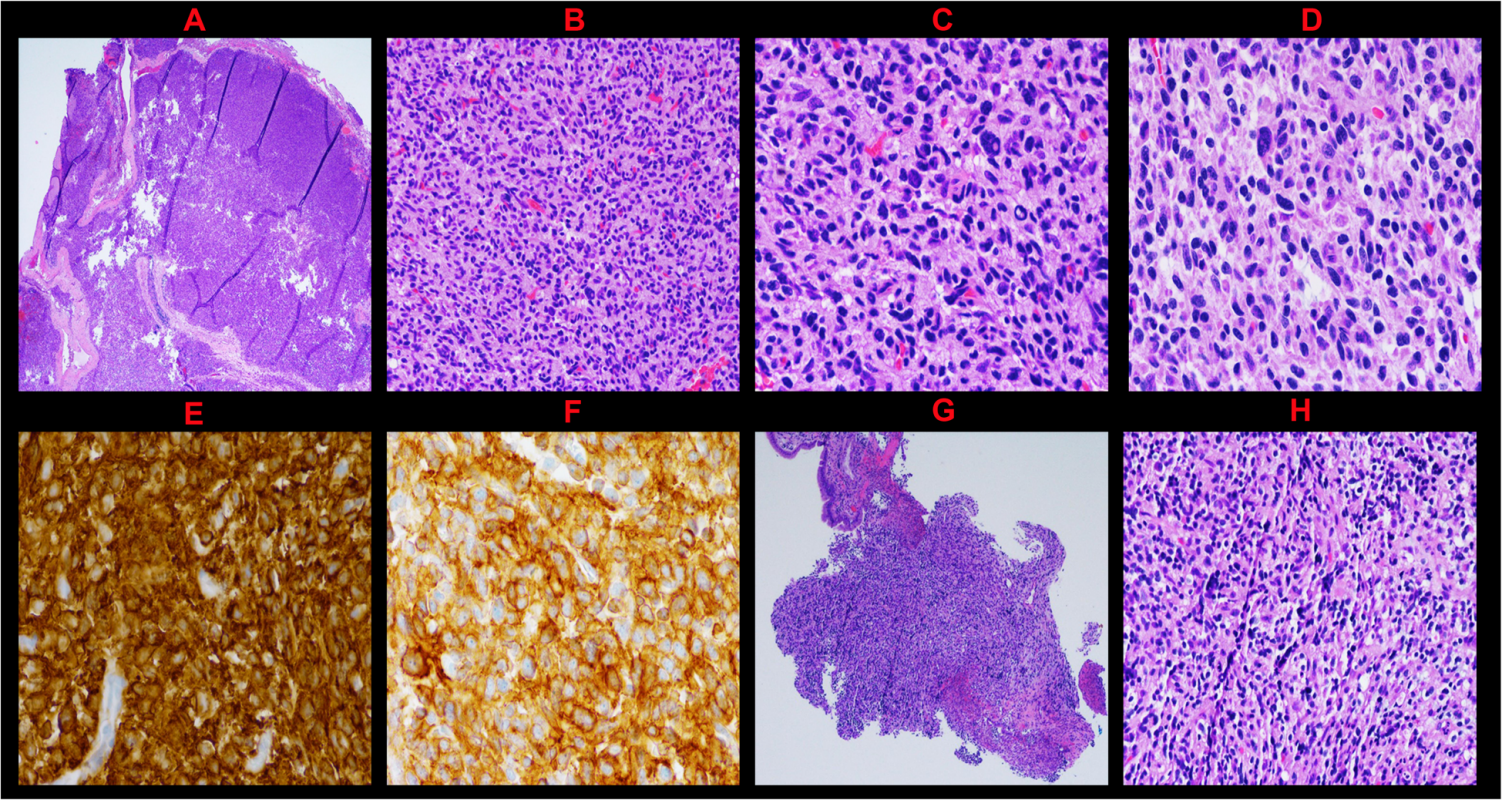
Pathology Slides. **a** Metastatic Intradural Tumor: The tumor is very cellular consisting of sheets of tumor cells (hematoxylin-eosin, original magnification X20). **b** Metastatic Intradural Tumor: The cells are haphazardly arranged and spindled with short, ovoid, irregular, hyperchromatic nuclei with scattered large atypical nuclei (hematoxylin-eosin, original magnification X200). **c**, **d** Metastatic Intradural Tumor: The cytoplasm is eosinophilic and moderate in amount with variable vacuolization. Occasional nuclear inclusions are seen. (hematoxylin-eosin, original magnification X400). **e**, **f** Metastatic Intradural Tumor: The tumor cells are diffusely positive for KIT (CD117) (**e**) and ANO-1 (DOG**-**1) (**f**) immunohistochemical stains (original magnification X400). **g**, **h** The original gastric biopsy shows a subepithelial tumor consisting of predominantly bland small epithelioid spindled cells with vacuolated cytoplasm and smudged chromatin. Rare large cells are noted, but not to the extent seen in the metastatic tumor (hematoxylin-eosin, original magnification X20 and X200, respectively)

The patient underwent left temporal craniotomy to excise the lesion without complication. Intraoperative pathology revealed a spindle cell neoplasm. Her post-operative course was uncomplicated; after an overnight stay in the ICU, the patient was transferred to the floor and discharged to a rehabilitation facility at her preoperative baseline. Postoperative MRI demonstrated gross total resection of the lesion. Microscopically, the tumor demonstrated sheets of spindled tumor cells with short ovoid, irregular, hyperchromatic nuclei and scattered large atypical nuclei without extensive necrosis (Fig. 2a-d). Immunohistochemical staining with appropriate controls was positive for KIT (Fig. 2e) and ANO1 (DOG1) (Fig. 2f), confirming diagnosis of metastatic GIST from the initial gastric tumor (Fig. 2g-h). She was subsequently re-initiated on imatinib (400 mg daily) as an outpatient following craniotomy.

## Results

**Fig. 3:**
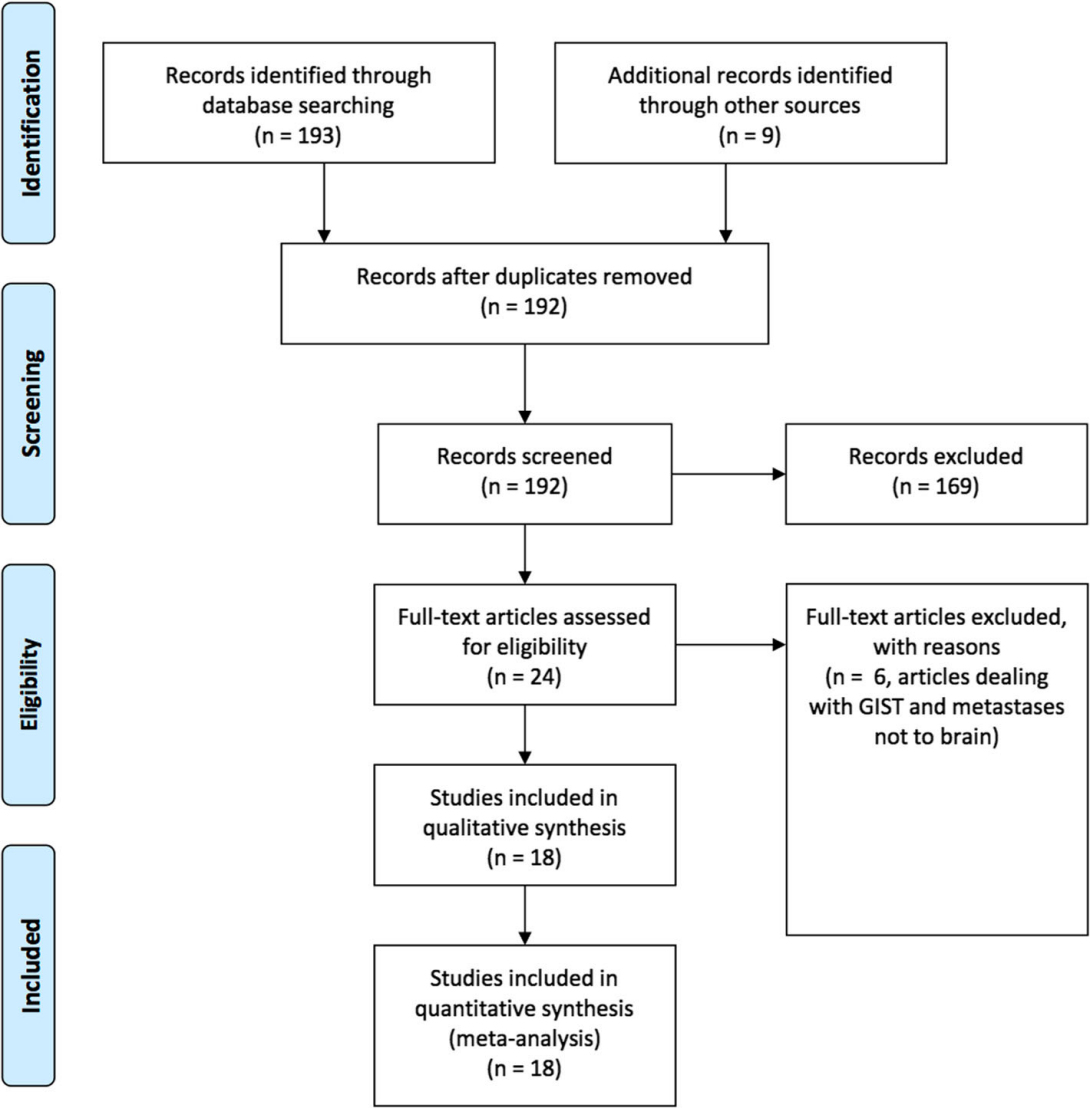
Systematic Review Flow Diagram

The initial database query yielded 192 publications after duplicates were removed. Screening of these publications based on abstracts yielded 24 publications, which was then further refined to 18 after the exclusion of 6 articles discussing GIST and metastases not to the brain (Fig. 3). These 18 articles were then included for review.

In our review of the literature, only 18 cases of GIST with intracranial metastases were identified, and these are summarized in Table 1. With the inclusion of our present case, 15 of 19 patients were male, and mean age was 58 years old (range 15–80 years, standard deviation 17.4 years). The primary site of the GIST in these patients was variable throughout the GI tract with the most common involving the small intestine (9 out of 19 cases) and the stomach (5 out of 19 cases). Additionally, although it was not reported in every case, any sites of intraperitoneal metastasis were also quite variable within this series with the most common site being the liver. With regards to sites of brain metastasis, there was a large predilection for the cerebrum, with only one case of metastasis solely to infratentorial elements, namely the pontomedullary junction and the cerebellum [7]. The tumors in seven of the cases, including the case reported here, either involved or originated from the dura [4, 8–12]. There was one metastasis to the pituitary [13]. Radiographically, the tumors reported tended to have appearances similar to other intracranial metastases, being relatively well-circumscribed and contrast-enhancing. Fourteen patients underwent chemotherapy (4 as stand-alone therapy, 10 in conjunction with other treatments), 13 patients had total resection (all in conjunction with other treatments), eight patients underwent radiation therapy (1 as stand-alone therapy, 7 in conjunction with other treatments), and four patients had stereotactic radiosurgery (all in conjunction with other treatments). Eight patients died following treatment of their intracranial disease. Of these, the average time to death was 9.6 months (range 3.5–35 months, standard deviation 10.6 months). These data are summarized in Table 1.

**Table 1:** Summary of Studies Included in Systematic Review

	Case	Age	Sex	Primary	CNS site	Size of CNS lesion	Other Mets	Interval between diagnosis and CNS metastasis	Treatment of CNS tumor	Mutation Status	Outcome (from time of CNS diagnosis)
1	Akiyama 2004 [8]	60	M	Small bowel	Left cavernous sinus	NR	L5-S1 vertebra	7 years	Radiation (54 Gy), not further described	NR	Death at 8 months
2	Badri 2018 [15]	66	M	Small Bowel	Right cerebellar hemisphere	4 cm	NR	CNS lesion found first	Total resection of cerebellar tumor with adjuvant radiation and chemotherapy (no further details given)	NR	Remission at 12 months
3	Brooks 2002 [4]	75	M	Mesentery	Both hemispheres, (dural based)	Infiltrative	Liver	14 months	Imatinib 400 mg twice daily	NR	Remission at 4 months
4	Drazin 2012 [16]	57	M	Stomach	Left cerebellar, left frontal	3 cm for cerebellar lesion, size of frontal lesion not provided	NR	13 months	Total resection for cerebellar lesions, SRS (18 Gy) to frontal lesion	NR	Remission at 15 months
5	Gerin 2007 [7]	45	M	Small bowel	Pontomedullary junction, cerebellum, leptomeninges	2 cm for primary lesion, others very small	NR	5 years	Imatinib 800 mg daily	NR	Death at “a few weeks”
6	Hamada 2010 [17]	54	F	Esophagus	Left frontal lobe	5 cm	Liver	6 years	Neoadjuvant imatinib 400 mg daily, then total resection, SRS (dose not reported)	KIT (exon 11)	Remission at 6 months
7	Hughes 2004 [9]	47	M	Jejunum	Left parasagittal (dural based)	NR	Liver	25 Months	Total resection, Imatinib 800 mg daily	KIT (exon 9)	Death at 35 months
8	Inage 2002 [10]	70	M	Stomach	Left occipital, (dural based)	5.5 cm	Lung	10 years	Total resection and radiation, not further described	NR	Death at 8 months
9	Jagannathan 2012 [11]	15	M	Stomach	Right Frontoparietal, (dural based)	4.2 cm × 3.3 cm × 3.1 cm	Liver	12 years	Many TK inhibitors prior to discovery of CNS lesion, Total resection	No mutation in KIT or PDGFR alpha	Remission at 6 months
10	Janku 2011 [18]	56	F	Stomach	Many small lesions	NR	Lung, liver, pelvis	3 weeks	Imatinib 400 mg daily, then 600 mg daily	NR	NR
11	Kajikawa 2005 [19]	76	M	Jejunum and Duodenum	Right parietal, Right cerebellar hemisphere	2 cm	NR	4 months	Imatinib 400 mg daily, radiation (WBRT 40 Gy)	No mutation in KIT	Death at 4 months
12	Kaku 2006 [12]	68	F	Perisacral	Right parietal lobe, (dural based)	3 cm	NR	2 years	Total resection, imatinib 800 mg daily	NR	Remission
13	Naoe 2011 [20]	77	M	Jejunum	Right cerebral peduncle, left occipital lobe	2.4 cm, 2.2 cm	NR	CNS lesion found first	Total resection, WBRT (39 Gy), imatinib 400 mg daily	No KIT mutation or PDGFR alpha	Death at 4 months
14	O’Halloran 2017 [13]	61	M	NR	Pituitary	1.5 cm × 3.5 cm × 2 cm	NR	NR	Total resection, SRS and imatinib not further described	NR	NR
15	Puri 2006 [21]	42	M	Mesentery	Right Parietal lobe	3.5 cm	NR	CNS lesion found first	Total resection, WBRT (60 Gy), imatinib 600 mg daily, multiple cytotoxic chemotherapy regimens	NR	Death at 10 months
16	Sato 2014 [22]	80	M	Small bowel	Cerebellar vermis, Right frontal lobe	4 cm	Cardiac apex, subclavian vessels	CNS lesion found first	Total resection, radiotherapy (22 Gy) not further described	NR	Death at 4 months
17	Takeuchi 2014 [5]	74	M	Jejunum	Right prefrontal gyrus	1.4 cm ×1.5 cm	Liver	6 years	Sunitinib 50 mg daily, SRS not further described	NR	Remission at 9 months
18	Wong 2011 [23]	26	M	Duodenum	Left frontotemporal	6.1 cm ×4.1 cm	Liver	6 years	Total resection, radiation not further described	NR	Remission at 4 months
19	Present case	57	F	Esophagus and stomach	Left temporal, (dural based)	2.9 cm × 3.1 cm × 3.4 cm.	Liver	6 months	Total resection, imatinib 400 mg daily	NR	Follow-up in progress

*CNS*, Central Nervous System; *Mets*, Metastases; *M*, Male; *F*, Female; *NR*, Not Reported; *TK*, Tyrosine Kinase; *PDGFR*, Platelet-derived growth factor receptor; *SRS*, Stereotactic radiosurgery; *WBRT*, Whole Brain Radiation Therapy

## Discussion and conclusion

Metastatic GIST to the brain is a rare clinical entity requiring unique considerations for treatment. Interestingly, there was substantial variation in the treatment of these patients, reflecting the lack of evidence-based guidelines for treatment of intracranial GISTs. Nevertheless, treatment regimens in reviewed papers generally include combinations of surgery, radiotherapy, and systemic chemotherapy with tyrosine kinase inhibitors, similar in concept to treatment of other intracranial metastases. Thirteen of nineteen patients reported here underwent treatment with tyrosine kinase inhibitor therapy, while the other six patients received no specific chemotherapy. Traditionally, treatment of advanced or metastatic GIST has been challenging due to the tumor’s resistance to cytotoxic chemotherapeutic agents [11]. With the identification of the KIT gene, the development of targeted tyrosine kinase inhibitors has improved the response to medical treatment. Indeed, the presence of KIT gene is positively correlated with susceptibility to tyrosine kinase inhibitor therapy [3]. One study noted an initial reduction in intracranial tumor burden with sunitinib treatment, but noted that several weeks after initiation of treatment, a new brain metastasis developed which was then successfully treated with radiation [5]. The patient was started on imatinib, but after it proved ineffective, was switched to sunitinib. Although imatinib is known to have poorer CNS penetration, any clear advantage of sunitinib over imatinib in the treatment of GIST metastatic to the CNS is unlikely to be demonstrated with so few cases. Nevertheless, sunitinib and other tyrosine kinase inhibitors that have better CNS penetration remain interesting targets for future research.

GIST, similar to the histologically-similar soft tissue sarcomas, is generally considered to be radioresistant. As such, radiation has been considered to have a limited role in treatment. Nevertheless, some series have reported at least a good palliative effect of radiotherapy for GIST metastases that are refractory to therapy or not amenable to surgery [14]. Eight patients in this series underwent radiation therapy of some kind. The use of conventional radiation versus radiosurgery was variable, as were the radiation doses when reported. Additionally, only one patient reported underwent radiation without additional therapy, in the case of a cavernous sinus metastasis.

Finally, surgery consisting of local control of intracranial disease with adjuvant therapy remains a useful treatment modality for metastatic GIST, especially with regards to intracranial metastases [3, 17, 18]. Intracranial surgery is especially reasonable when metastasis is small, localized, and superficial (cortical/subcortical). Nevertheless, many case series and reports describe cases of metastatic GIST with good response to radiation or chemotherapy alone. Given these other reports of tumors responsive to chemotherapy and radiation, it seems more investigation is required to determine the best course of treatment for patients with this unusual sequela of GIST.

## Data Availability

The datasets used and/or analyzed during the current study are available from the corresponding author on reasonable request.

## Abbreviations

c-KIT: CD 117
GIST: Gastrointestinal Stromal Tumors
PRISMA: Preferred Reporting Items for Systematic Reviews and Meta-Analyses

## References

[1] Davila Raquel E., Faigel Douglas O. (2003). GI stromal tumors. Gastrointestinal Endoscopy.

[2] Patil DT, Rubin BP (2011). Gastrointestinal stromal tumor: advances in diagnosis and management. Arch Pathol Lab Med.

[3] Tanaka T. Gastrointestinal Stromal Tumors with Intracranial Metastasis: Treatment Strategy and Review of the Literature. Brain Metastases from Primary Tumors. 3: Elsevier; 2016. p. 213–224.

[4] Brooks B, Bani J, Fletcher C, Demeteri G (2002). Response of metastatic gastrointestinal tumor including CNS involvement to Imatinib Mesylate. J Clin Oncol.

[5] Takeuchi H, Koike H, Fujita T, Tsujino H, Iwamoto Y (2013). Sunitinib treatment for multiple brain metastases from Jejunal gastrointestinal stromal tumor: case report. Neurol Med Chir.

[6] Moher D, Liberati A, Tetzlaff J, Altman DG, Group P (2009). PLoS Med.

[7] Gerin F, Baloglu O, Morgan JA, Kesari S (2007). Central nervous system metastases from imatinib mesylate resistant gastrointestinal stromal tumor. J Neuro-Oncol.

[8] Akiyama K, Numaga J, Kagaya F, Takazawa Y, Suzuki S, Koseki N (2004). Case of optic nerve involvement in metastasis of a gastrointestinal stromal tumor. Jpn J Ophthalmol.

[9] Hughes B, Yip D, Goldstein D, Waring P, Beshay V, Chong G (2004). Cerebral relapse of metastatic gastrointestinal stromal tumor during treatment with imatinib mesylate: case report. BMC Cancer.

[10] Inage Y, Yamabe K, Yamamoto T, Sato Y, Ishikawa S, Onizuka M (2002). Resection for pulmonary metastasis of gastrointestinal stromal tumor of the stomach at 10 years after gastrectomy; report of a case. Kyobu Geka.

[11] Jagannathan J, Ramaiya N, Shinagare A, Hornick J, George S (2012). Intracranial Mestastasis from pediatric GI stromal tumor. J Clin Oncol.

[12] Kaku S, Tanaka T, T O, Seki K, Sawauchi S, Numoto R, et al. Perisacral Gastrointestinal Stromal Tumor with Intracranial Metastasis Neurol Med Chir 2006;46:254–257. 10.2176/nmc.46.254 16723820

[13] O'Halloran PJ, Hannon AM, Bartels C, McCawley N, Agha A, Brett F (2017). Gastrointestinal stromal tumor metastases to the pituitary: a rare entity. Br J Neurosurg.

[14] Cuaron J, Goodman K, Lee N, Wu A (2013). External beam radiation therapy for locally advanced and metastatic gastrointestinal stromal tumors. Radiat Oncol.

[15] Badri M, Chabaane M, Gader G, Bahri K, Zammel I (2018). Cerebellar metastasis of gastrointestinal stromal tumor: a case report and review of the literature. Int J Surg Case Rep.

[16] Drazin D, Spitler K, Jeswani S, Shirzadi A, Bannykh S, Patil C (2013). Multiple intracranial metastases from a gastric gastrointestinal stromal tumor. J Clin Neurosci.

[17] Hamada S, Itami A, Watanabe G, Nakayama S, Tanaka E, Hojo M (2010). Intracranial metastasis from an esophageal gastrointestinal stromal tumor. Intern Med.

[18] Janku F, Kidney D, Coyne J (2011). Unusual presentation of gastrointestinal stromal tumor with early cerebral involvement. Ir J Med Sci.

[19] Kajikawa M, Ishiyama A, Sawada K, Ono K, Suzuki Y (2005). Multiple gastrointestinal stromal tumors of duodenum and jejunum accompanied by lymph node and brain metastasis: report of a case. Japanese J Gastroenterol Surg.

[20] Naoe H, Kaku E, Ido Y, Gushima R, Maki Y, Saito H (2011). Brain metastasis from gastrointestinal stromal tumor: a case report and review of the literature. Case Rep Gastroenterol.

[21] Puri T, Gunabushanam G, Malik M, Goyal S, Das AK, Julka PK (2006). Mesenteric gastrointestinal stromal tumour presenting as intracranial space occupying lesion. World J Surg Oncol.

[22] Sato K, Tanaka T, Kato N, Ishii T, Terao T, Murayama Y (2014). Metastatic cerebellar gastrointestinal tumor with obstructive hydrocephalus arising from the small Intestsine: a case report and review of the literature. Case Rep Oncol Med.

[23] Wong CS, Chu YC (2011). Intra-cranial metastasis of gastrointestinal stromal tumor. Chin Med J.

